# The Influence of Arthritis in Other Major Joints and the Spine on the One-Year Outcome of Total Hip Replacement: A Prospective, Multicenter Cohort Study (EUROHIP) Measuring the Influence of Musculoskeletal Morbidity

**DOI:** 10.2106/JBJS.16.01040

**Published:** 2017-09-06

**Authors:** Joerg Huber, Paul Dieppe, Karsten Dreinhoefer, Klaus-Peter Günther, Andrew Judge

**Affiliations:** 1 Department of Orthopedics, Stadtspital Triemli, Zurich, Switzerland; 2 University of Exeter Medical School, University of Exeter, Exeter, United Kingdom; 3 Centre of Musculoskeletal Surgery, Charité, University of Berlin, Berlin, Germany; 4 Department of Orthopaedics, Traumatology and Sports Medicine, Medical Park Berlin Humboldtmuehle, Berlin, Germany; 5 University Center of Orthopaedics and Traumatology, Gustav Carus University Medicine, Technical University Dresden, Dresden, Germany; 6 Nuffield Department of Orthopaedics, Rheumatology and Musculoskeletal Sciences, University of Oxford, Headington, United Kingdom; 7 MRC Lifecourse Epidemiology Unit, Southampton General Hospital, University of Southampton, Southampton, United Kingdom

## Abstract

**Background:**

Although arthritis in other affected major joints and back pain are known to lead to worse outcomes following total hip replacement, to our knowledge, these risk factors have not previously been operationalized as a musculoskeletal morbidity profile. The aim of this study was to measure the influence of other major joints and the spine (as grades of musculoskeletal morbidity) on the 1-year outcome of primary total hip replacement.

**Methods:**

The EUROHIP study consists of 1,327 patients undergoing primary total hip replacement for arthritis across 20 European orthopaedic centers. The primary outcome was the responder rate at 12 months calculated with the relative effect per patient for total hip replacement using the total Western Ontario and McMaster Universities Osteoarthritis Index (WOMAC) score. The primary predictor of interest was different combinations of arthritis of major joints and the spine grouped into 4 musculoskeletal morbidity grades: 1 (single major joint), 2 (multiple major joints), 3 (single major joint and spine), and 4 (multiple major joints and spine). The confounders adjusted for were age, sex, body mass index, living situation, years of hip pain, American Society of Anesthesiologists (ASA) class, anxiety or depression, and preoperative WOMAC subscales.

**Results:**

For this analysis, 845 patients were included with complete 12-month follow-up WOMAC scores. The mean patient age was 65.7 years, and 55.2% of patients were female.

**Conclusions:**

The involvement of other major joints and the spine assessed as 1 of 4 musculoskeletal morbidity grades had a strong influence on the 1-year outcome after total hip replacement. The effect size was large compared with other risk factors. Even so, the majority of patients in musculoskeletal morbidity grade 4 had favorable outcomes from the surgical procedure (>74% response to surgical procedures).

**Level of Evidence:**

Therapeutic Level IV. See Instructions for Authors for a complete description of levels of evidence.

Even if primary total hip replacement might be considered today as a standardized surgical procedure, the outcome varies from patients with no more symptoms and/or disability after total hip replacement to those with some symptoms or residual disability and those with even more symptoms or disability. As a result of this residual disability in some patients, the mean Western Ontario and McMaster Universities Osteoarthritis Index (WOMAC) score in the EUROHIP cohort 1 year after total hip replacement was 15% rather than 0%. Other studies have confirmed this variation with positive responder rates for primary total hip replacement from 84% to 93%^1-5^. Predictors of worse outcome are older age, low symptom or disability score, high body mass index (BMI), higher number of general comorbidities, musculoskeletal morbidity of major joints and the spine, and depression^3,4,6-17^.

Although it is well known that musculoskeletal morbidity can have a negative impact on the overall outcome^6,11,14^, it is still not clear how large its influence is. Hawker et al. found that 81.2% of other large joints (knees and the contralateral hip) were troublesome in a cohort with hip arthritis coming for total hip replacement, and only half of these patients achieved a good outcome^11^. In a large multicenter study of total hip replacement, Quintana et al. described a high prevalence of contralateral hip arthritis (42.9%) and back pain (54.5%) with less improvement on some of the Short Form-36 (SF-36) and WOMAC domains in such patients 6 months after total hip replacement^14^. Ayers et al. found coexisting pain in the lumbar spine and other nonoperatively treated joints to be an important confounder for outcome after knee replacement and described the need for a Musculoskeletal Comorbidity Index^18^.

There are a limited number of possibilities for grading the severity of musculoskeletal comorbidities by focusing on functional limitations, as proposed by Charnley^19^ and Katz et al.^20^. Charnley differentiated the patients coming for total hip replacement into 3 groups depending on estimated factors that may limit the walking capacity (the affected hip as the only factor in the first group, both hips affected as factors in the second group, and other or unknown factors in the third group). Katz et al. proposed a score of musculoskeletal functional limitations as the sum of limitations in 6 separate anatomic regions (knee; hip; back; hand, wrist, arm, and shoulder; foot and ankle; and neck). Neither approach included the combinations of different affected major joints and the spine together as a grade of musculoskeletal morbidity.

The objective of this study was to measure the influence of other major joints and the spine (as a grade of musculoskeletal morbidity) on the outcome 1 year after total hip replacement in a large European multicenter cohort (EUROHIP). The null hypothesis was that musculoskeletal morbidity does not influence total hip replacement outcome.

## Materials and Methods

### Study Design

**Fig. 1:**
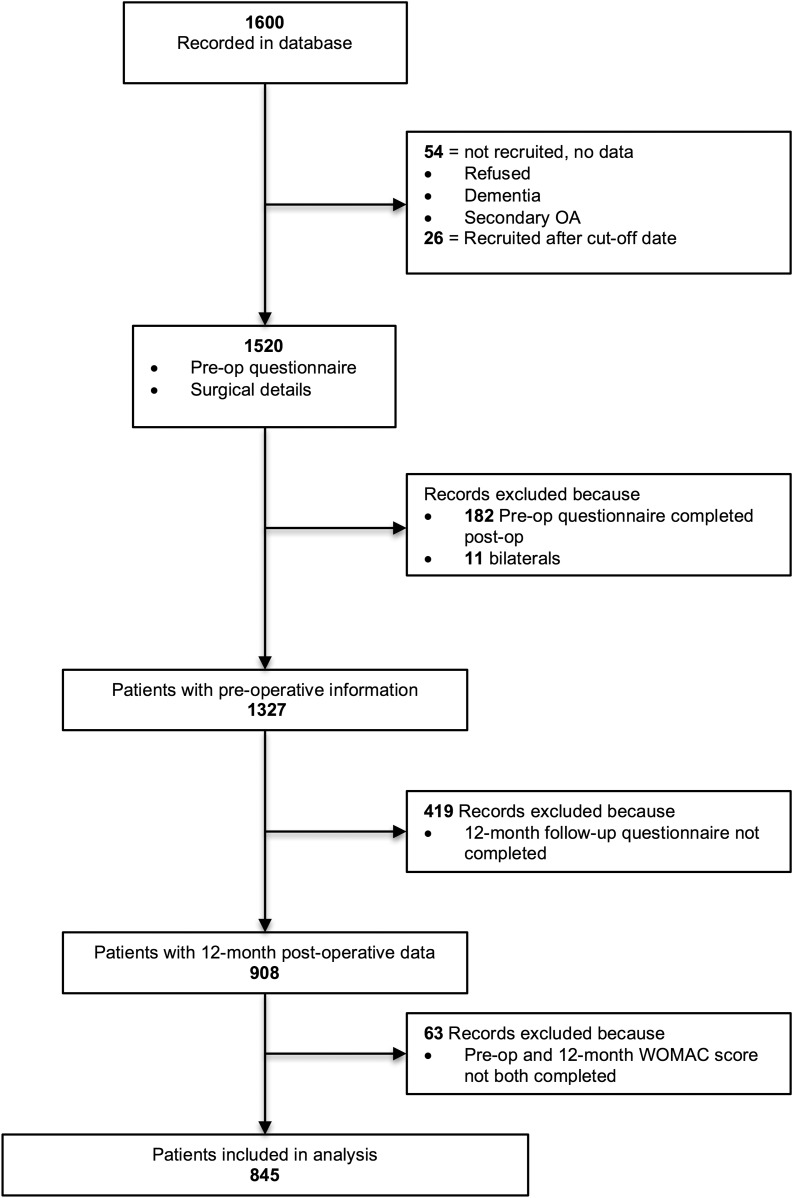
Flowchart for the EUROHIP study. OA = osteoarthritis.

The EUROHIP study included 1,327 patients undergoing primary total hip replacement across 20 European orthopaedic centers in 12 nations^21^. It began collecting data in January 2004 and concluded in December 2006. Inclusion criteria were a diagnosis of primary hip arthritis, primary total hip replacement, and signed informed consent. Primary arthritis of the hip was defined as symptomatic hip disease with radiographic evidence of arthritis and no obvious predisposing cause such as unequivocal dysplasia, congenital dislocation of the hip, Legg-Calvé-Perthes disease, or osteonecrosis. Exclusion criteria comprised trauma, severe mental illness or dementia, patient unwillingness or inability to participate, and unequivocal evidence of secondary arthritis. Each center was responsible for local ethical approval. The study protocol and data collection forms were designed in Bristol, United Kingdom, and Ulm, Germany, by the study principal investigators and the study coordinator. The patient questionnaire was reviewed for acceptability in Bristol and modified accordingly before being sent to Ulm for translation and distribution. Questionnaires were sent to each center for translation and were returned for editing before printing and distribution with a set of instructions. In this study, 845 patients were included (Fig. 1) with complete follow-up of patient-reported outcome measures (WOMAC) before total hip replacement and at 1 year postoperatively.

### Outcome

Patients completed a WOMAC questionnaire prior to the surgical procedure and at the 12-month follow-up^22^. This questionnaire consists of 24 items in 3 subscales: pain (5), stiffness (2), and physical function (17). For each subscale, a normalized score was created (0 indicating no symptoms and 100 indicating extreme symptoms) by summing up the total score of each subscale, multiplying it by 100, and dividing by the maximum score. A total score of 96 was created by combining the 3 subscales and then was converted into a normalized score.

The relative effect per patient ([pretreatment score – posttreatment score]/pretreatment score)^2,23^ was calculated for each patient using the total WOMAC score. A relative effect per patient of 1 (best score) corresponds to a patient without symptoms or disability after treatment; a relative effect per patient of 0.5, to 50% reduction; a negative relative effect per patient, to more symptoms or disability at follow-up; and −0.5, to a worsening of 50%.

### Main Predictor

The primary predictor of interest was the influence of other joints and the spine. Prior to the surgical procedure, patients were asked whether they had arthritis in any other parts of their body including large joints (shoulder, elbow, or hand; contralateral hip; knee; ankle or foot) and the spine (neck; lower back). All patients can be differentiated into 4 grades of musculoskeletal morbidity (Table I): grade 1 includes a single joint (in this case, only the index hip joint); grade 2 includes multiple major joints (the index hip joint and ≥1 other major joints); grade 3 includes a single joint (the index hip joint) and the spine; and grade 4 includes multiple major joints (the index hip joint and ≥1 other major joints) and the spine.

**TABLE I:** Description of Musculoskeletal Morbidity Grades

Description	Grade	No. of Patients*
Index joint, without spine	1	416 (32.1%)
Index and other major joints, without spine	2	479 (36.9%)
Index joint, with spine	3	112 (8.6%)
Index and other major joints, with spine	4	291 (22.4%)

* Data were missing for 29 patients, leaving 1,298 patients to be evaluated.

### Confounders

Prior to the surgical procedure, patients completed questionnaires including a wide range of demographic information. Demographic information considered relevant in this study included age, sex, BMI, whether or not the patient lives alone or with someone else, and the number of years that the patient has had hip pain. Surgical teams recorded information on the patient’s American Society of Anesthesiologists (ASA) class (scored from 1 [normal, healthy] to 4 [life-threatening systemic disease]). Information on anxiety or depression was taken from the EuroQol-5 Dimensions (EQ-5D) questionnaire subscale. Preoperative WOMAC subscales of pain, stiffness, and function were included as further potential confounders.

### Statistical Methods

Descriptive statistics (the mean and standard deviation for continuous variables, and the number and percentage for categorical variables) were used to describe the characteristics of patients within the 4 musculoskeletal morbidity groups. A box-and-whisker plot was used to graphically describe the overall relative effect per patient score within each of the 4 musculoskeletal morbidity groups.

Logistic regression modeling was used to describe the association of the main predictor (musculoskeletal morbidity groups) with the outcome of interest (responder rate according to the relative effect per patient score), controlling for confounding variables. The results of the regression model are presented as relative risk ratios by fitting a generalized linear model with a binomial error structure and a log link function (log-logistic model). Fractional polynomial regression was used to assess evidence of linearity of associations of continuous predictors with the outcome. Multiple imputation by chained equations was used to account for the cumulative effect of missing data in several of the variables^24^. Forty imputed data sets were generated using all potential factors (including the outcome), and estimated parameters were combined using Rubin’s rules^25^.

## Results

The characteristics of patients who completed the 12-month follow-up questionnaire (n = 845) were similar to those of patients in the whole sample (n = 1,327) (Table II). Patients with only baseline assessment (n = 482) (i.e., patients lost to follow-up) were more likely to be living alone and had higher levels of anxiety or depression.

**TABLE II:** Descriptive Characteristics and Comparison of Patients with Only Baseline Assessment and with Complete Follow-up*

Variable	Baseline Only (N = 482)	Complete Follow-up (N = 845)	All Patients (N = 1,327)
Musculoskeletal morbidity grade†			
1	141 (30.5%)	275 (32.9%)	416 (32.1%)
2	167 (36.1%)	312 (37.4%)	479 (36.9%)
3	55 (11.9%)	57 (6.8%)	112 (8.6%)
4	100 (21.6%)	191 (22.9%)	291 (22.4%)
Age‡ *(yr)*	65.7 ± 11.3	65.7 ± 10.6	65.7 ± 10.9
Sex†			
Male	200 (43.0%)	359 (44.8%)	559 (44.1%)
Female	265 (57.0%)	443 (55.2%)	708 (55.9%)
BMI‡ *(kg/m*^*2*^*)*	27.0 ± 4.3	27.8 ± 4.4	27.5 ± 4.4
Living situation†			
Alone	134 (28.3%)	207 (24.6%)	341 (25.9%)
With spouse or partner	309 (65.2%)	591 (70.1%)	900 (68.3%)
With somebody else	31 (6.5%)	45 (5.3%)	76 (5.8%)
Anxiety or depression†			
None	198 (47.6%)	500 (59.9%)	698 (55.8%)
Moderate	187 (45.0%)	309 (37.0%)	496 (39.7%)
Extreme	31 (7.5%)	26 (3.1%)	57 (4.6%)
Years of hip pain†			
<1	57 (12.0%)	84 (10.0%)	141 (10.7%)
1 to 2	132 (27.9%)	242 (28.8%)	374 (28.5%)
3 to 5	148 (31.2%)	255 (30.4%)	403 (30.7%)
>5	137 (28.9%)	259 (30.8%)	396 (30.1%)
ASA class†			
1	92 (21.1%)	117 (15.8%)	209 (17.8%)
2	250 (57.3%)	469 (63.5%)	719 (61.2%)
3 or 4	94 (21.6%)	153 (20.7%)	247 (21.0%)
Preoperative WOMAC scores‡ *(points)*			
Pain	57.9 ± 18.0	54.2 ± 17.6	55.4 ± 17.8
Stiffness	60.5 ± 22.0	60.5 ± 20.1	60.5 ± 20.7
Function	63.3 ± 16.8	58.6 ± 16.5	60.1 ± 16.7

* Data were missing for the following patients: 29 patients (2.2%) in both the musculoskeletal morbidity grade and age categories; 60 patients (4.5%) in the sex category; 102 patients (7.7%) in the BMI category; 10 patients (0.8%) in the living situation category; 76 patients (5.7%) in the anxiety or depression category; 13 patients (1.0%) in the years of hip pain category; 152 patients (11.5%) in the ASA class category; and, in the preoperative WOMAC score categories, 72 (5.4%) for pain, 61 (4.6%) for stiffness, and 74 (5.6%) for function.

† The values are given as the number of patients with data available, with the percentage in parentheses.

‡ The values are given as the mean and the standard deviation.

Of the 845 patients included in this analysis, the mean age was 65.7 years (range, 26 to 92 years), and 55.2% of patients were female. One-quarter of patients lived alone and 59.9% reported no symptoms of anxiety or depression. The majority of patients (90%) had symptoms of hip pain for >1 year prior to the surgical procedure, with 30.8% of these patients having symptoms for >5 years. Regarding the pattern of musculoskeletal morbidity, 32.9% had hip arthritis in the index joint only (grade 1), 37.4% had arthritis in multiple major joints (grade 2), 6.8% had hip arthritis in the index joint in addition to spinal arthritis (grade 3), and 22.9% had arthritis in multiple major joints in addition to spinal arthritis (grade 4).

**Fig. 2:**
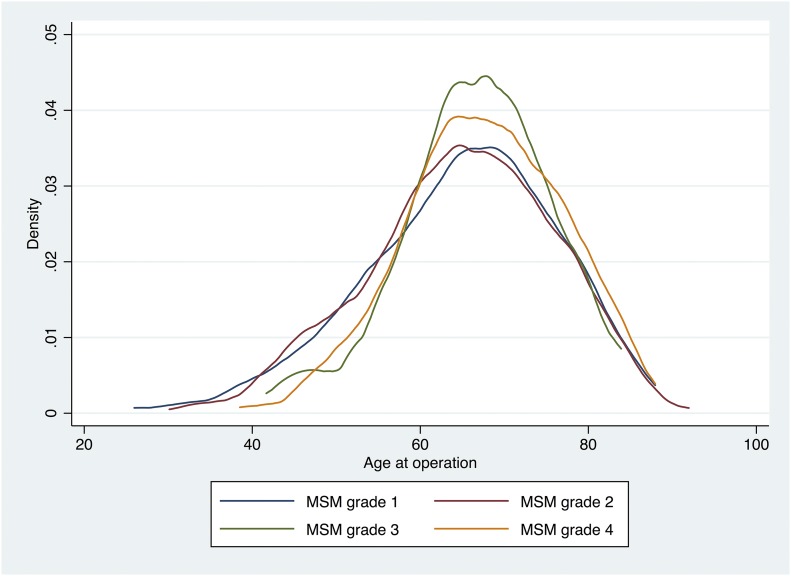
Kernel density plot describing distribution of age within each musculoskeletal morbidity (MSM) grade.

The characteristics of patients within each of the 4 musculoskeletal morbidity groups are described in Table III. Patients with spinal pathology were slightly older compared with those with arthritis in other major joints, but, overall, the distribution of age was very similar across all morbidity groups (Fig. 2). Patients with arthritis in multiple major joints (grades 2 and 4) were more likely to be female, to live alone, and to have had hip pain for a greater number of years prior to the surgical procedure. Anxiety or depression was more common in those with spinal arthritis (grades 3 and 4). There were fewer patients with ASA class 3 or 4 in the group with musculoskeletal morbidity grade 1 (arthritis only in the index hip) at 12%, compared with the group with musculoskeletal morbidity grade 4 (spinal arthritis and arthritis in multiple major joints) at 30%. No differences were observed in preoperative WOMAC subscales across the morbidity groupings.

**TABLE III:** Characteristics of Patient Subgroups in the Musculoskeletal Morbidity Grades

	Musculoskeletal Morbidity Grade			
Characteristic	1	2	3	4
Age* *(yr)*	64.9 ± 11.4	64.9 ± 10.9	66.8 ± 8.8	67.6 ± 9.3
Sex†				
Male	127 (48.7%)	130 (43.6%)	25 (47.2%)	72 (40.0%)
Female	134 (51.3%)	168 (56.4%)	28 (52.8%)	108 (60.0%)
BMI* *(kg/m*^*2*^*)*	27.5 ± 4.5	28.2 ± 4.6	26.3 ± 3.3	27.8 ± 4.3
Living situation†				
Alone	59 (21.5%)	74 (23.7%)	12 (21.1%)	59 (31.1%)
With spouse or partner	201 (73.4%)	222 (71.2%)	44 (77.2%)	118 (62.1%)
With somebody else	14 (5.1%)	16 (5.1%)	1 (1.8%)	13 (6.8%)
Anxiety or depression†				
None	171 (63.1%)	187 (60.9%)	31 (55.4%)	106 (55.5%)
Moderate	94 (34.7%)	108 (35.2%)	23 (41.1%)	79 (41.4%)
Extreme	6 (2.2%)	12 (3.9%)	2 (3.6%)	6 (3.1%)
Years of hip pain†				
<1	36 (13.1%)	31 (9.9%)	6 (10.5%)	11 (5.8%)
1 to 2	81 (29.5%)	96 (30.8%)	18 (31.6%)	46 (24.3%)
3 to 5	79 (28.7%)	85 (27.2%)	21 (36.8%)	67 (35.5%)
>5	79 (28.7%)	100 (32.1%)	12 (21.1%)	65 (34.4%)
ASA class†				
1	47 (19.4%)	46 (17.1%)	10 (19.2%)	14 (8.3%)
2	166 (68.6%)	162 (60.2%)	31 (59.6%)	104 (61.9%)
3 or 4	29 (12.0%)	61 (22.7%)	11 (21.2%)	50 (29.8%)
Preoperative WOMAC scores* *(points)*				
Pain	51.4 ± 18.2	54.8 ± 17.3	53.3 ± 16.0	57.7 ± 17.3
Stiffness	58.1 ± 19.9	62.2 ± 19.5	60.3 ± 19.8	61.2 ± 20.9
Function	55.6 ± 16.9	59.2 ± 15.9	59.2 ± 15.6	62.1 ± 16.6

* The values are given as the mean and the standard deviation.

† The values are given as the number of patients with available data, with the percentage in parentheses.

**Fig. 3:**
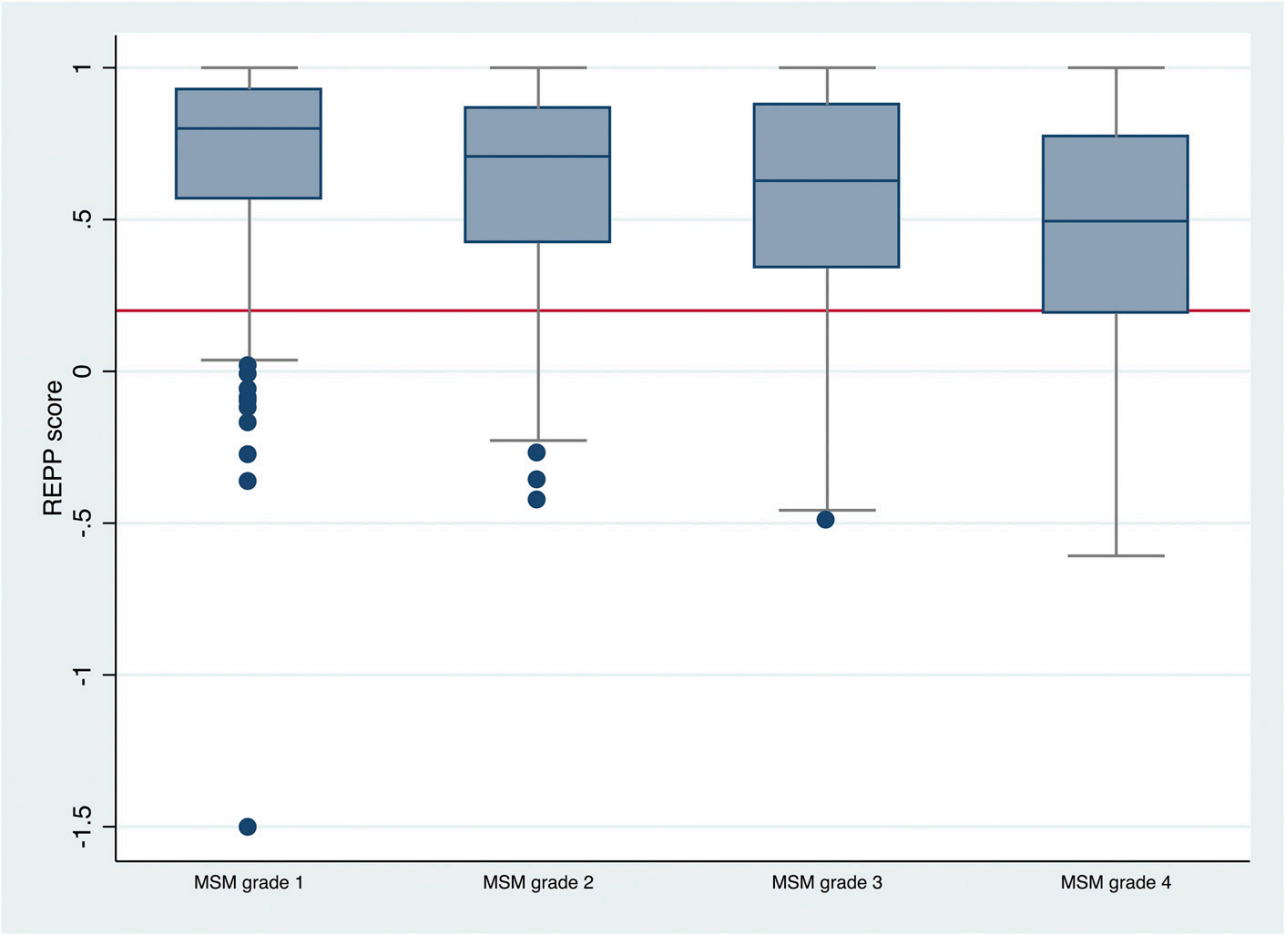
Box-and-whisker plot describing the relative effect per patient (REPP) score with each musculoskeletal morbidity (MSM) grade. The whiskers indicate the interquartile range and the length of the whiskers indicates the 1.5 times interquartile range. The red line indicates a REPP score of 0.2; patients with a REPP score of >0.2 are considered as responders who were favorable to the surgical procedure.

As the grade of musculoskeletal morbidity increased, the outcome according to the relative effect per patient score declined (Fig. 3). Those with musculoskeletal morbidity grade 1 had the best outcome, and those with musculoskeletal morbidity grade 4 had the worst outcome.

**Fig. 4:**
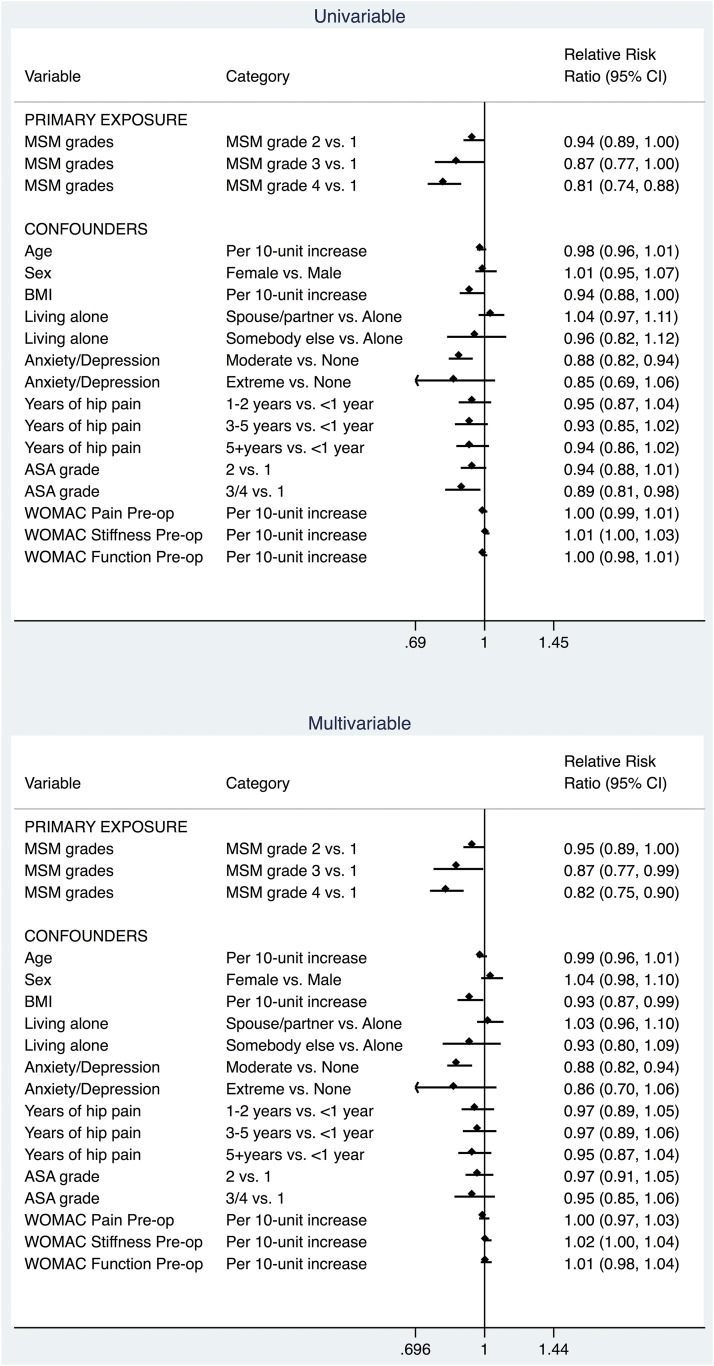
Forest plot describing the results of univariable and multivariable logistic regression models. CI = confidence interval and MSM = musculoskeletal morbidity.

The findings of the logistic regression model confirmed an important significant association of grade of musculoskeletal morbidity with patient outcomes of total hip replacement surgical procedures (p < 0.001). As the grade of musculoskeletal morbidity increased, patients were less likely to achieve a favorable response to total hip replacement. The proportion of patients responding favorably to the surgical procedure was 74% for musculoskeletal morbidity grade 4 compared with 92% for musculoskeletal morbidity grade 1. After adjusting for a wide range of confounding factors, the risk of positive response remained 18% lower for musculoskeletal morbidity grade 4 compared with grade 1 (relative risk ratio, 0.82 [95% confidence interval, 0.75 to 0.90]) (Fig. 4). Hence, the null hypothesis was rejected.

## Discussion

The grade of musculoskeletal morbidity influences the outcome of total hip replacement; in this study, it had the largest effect of all compared parameters. The strengths of this study were the large number of participants and the generalizability, with participants from 20 different centers across Europe, different cultural regions, and 8 languages.

The model of 4 grades of musculoskeletal morbidity covers all different clinical situations of patients with hip arthritis undergoing total hip replacement. Using this differentiation should allow the surgeon to manage the patients’ expectations better, especially in patients with higher grades of musculoskeletal morbidity. These patients with a higher grade of musculoskeletal morbidity can present in the follow-up with new symptoms deriving from other joints and/or the spine. Therefore, it seems important to focus not only on the affected hip joint but also on the other major joints and the spine, which can influence the outcome. A failure to provide this clarification to the patient is likely a key source of patient dissatisfaction.

This universal model of affected major joints and the spine covers all of the various clinical situations of patients with arthritis in different major joints undergoing a replacement surgical procedure, from the simple case with only 1 affected major joint to the complex case with multiple other major joints affected and spinal abnormalities. This grading can also be applied for bilateral arthritis of major joints (hip, knee, or shoulder); this allows grouping of these patients in homogeneous subgroups with respect to outcome. Knee arthritis is known to frequently occur bilaterally (musculoskeletal morbidity grade 2), which can influence the outcome after any treatment. The use of this system for patients with arthritis of the knee or shoulder needs to be tested in other external cohorts of patients, as our data here are only for patients undergoing hip replacement surgical procedures.

An unexpected finding was the high prevalence of patients with musculoskeletal morbidity grades 2, 3, and 4 (68%); in other words, only one-third of the patients had just arthritis of the index joint. A second unexpected finding was that there were no differences in the sex and age distributions of patients in each of the 4 musculoskeletal morbidity grades. From the literature, we expected a higher mean age in the patient groups with a musculoskeletal morbidity grade of >1^11^. These findings need further study.

Using the 4 musculoskeletal morbidity grades in daily practice allows practitioners to counsel the patients better preoperatively and to manage their expectations of outcome with higher precision. Even patients with hip arthritis and musculoskeletal morbidity grade 4 profit from a surgical procedure with a responder rate of 75%, but the score after total hip replacement remains higher compared with musculoskeletal morbidity grade 1. In difficult, unclear situations, a test infiltration of the affected hip with local anesthetics may illustrate the potential effect of total hip replacement for the patient.

Bellamy et al. designed the WOMAC questionnaire to measure arthritis of 1 hip or knee (musculoskeletal morbidity grade 1). One-third of the patients in the current study fulfilled this criterion and were properly assessed^22^. Knowing this fact, we realized retrospectively that, in two-thirds of patients, the WOMAC is capturing additional symptoms or disability from other joints and/or the spine. A basic difficulty might be the lack of localization of symptoms in the WOMAC. New patient questionnaires have integrated the localization of symptoms for the patient as a whole: for example, Pationnaire and Intermittent and Constant Osteoarthritis Pain (ICOAP) (both mannequin-based systems)^26-29^.

The current study had several limitations. All participating centers had experience and interest in total hip replacement and therefore a positive selection bias of the included patients has to be supposed. Therefore, the centers were focused on total hip replacement and good outcome, which may lead to better results and a higher responder rate than in daily clinical practice. A limitation of the study was that it is not possible to clearly separate ipsilateral double arthritis (of the hip and knee) from ipsilateral hip and contralateral knee arthritis. Another limitation was the problem of ipsilateral hip and knee arthritis, in which the principal symptoms can present in the thigh and more distally. In this study, there were no special guidelines for these patients regarding further diagnosis of arthritis of the ipsilateral knee. There was no information about complications during the study or after total hip replacement and the further management of these patients. Therefore, an individual clinic might have excluded such patients from the study on the basis of the need for subsequent surgical procedures such as revision arthroplasty, while other such patients were included in the study because of a lack of awareness of the subsequent procedures. In addition, the grading of musculoskeletal morbidity depended on an additional question tested and validated locally in Bristol by only 1 coauthor. The distributions of the musculoskeletal morbidity grades showed no substantial differences across the participating centers.

In conclusion, arthritis in other major joints and the spine measured as grade of musculoskeletal morbidity has a strong influence on the 1-year outcome after total hip replacement. In this study, compared with other risk factors (anxiety or depression, low preoperative WOMAC score, female sex, and older age), arthritis in other major joints and the spine had the largest impact on outcome. The favorable response rates to hip arthroplasty declined stepwise with each grade of musculoskeletal morbidity (grade 1 [single joint] > grade 2 [multiple joints] > grade 3 [single joint and spine] > grade 4 [multiple joints and spine]). The prevalence of musculoskeletal morbidity grades 2, 3, and 4 in patients with arthritis of 1 hip was higher than expected (68% of the cohort). Even patients in musculoskeletal morbidity grade 4 still profited from a surgical procedure (>74% responder rate).
